# Development of the web-based Spanish and Catalan versions of the Euroqol 5D-Y (EQ-5D-Y) and comparison of results with the paper version

**DOI:** 10.1186/s12955-015-0271-z

**Published:** 2015-06-03

**Authors:** Noemí Robles, Luis Rajmil, Dolors Rodriguez-Arjona, Marta Azuara, Francisco Codina, Hein Raat, Ulrike Ravens-Sieberer, Michael Herdman

**Affiliations:** Agència de Qualitat i Avaluació Sanitàries de Catalunya (AQuAS), Roc Boronat 81-95 2nd Floor, Barcelona, 08005 Spain; Red de Investigación en Servicios de Salud en Enfermedades Crónicas (REDISSEC), Madrid, Spain; IMIM (Institut Hospital del Mar de Recerca Biomèdica), Barcelona, Spain; Centro de Investigación Epidemiológica en Red (CIBERESP), Madrid, Spain; Corporació de Salut del Maresme i la Selva, Calella, Spain; Department of Public Health, Erasmus MC – University Medical Center Rotterdam, Rotterdam, The Netherlands; Department of Child and Adolescent Psychiatry, Psychotherapy, and Psychosomatics, University Medical Center Hamburg- Eppendorf, Hamburg, Germany; Office of Health Economics, London, UK

**Keywords:** Agreement web and paper versions, Children and adolescents, Digital version, Health related quality of life, Reliability, Validity

## Abstract

**Background:**

The objectives of the study were to develop web-based Spanish and Catalan versions of the EQ-5D-Y, and to compare scores and psychometric properties with the paper version.

**Methods:**

Web-based and paper versions of EQ-5D-Y were included in a cross-sectional study in Palafolls (Barcelona), Spain and administered to students (n = 923) aged 8 to 18 years from 2 primary and 1 secondary school and their parents. All students completed both the web-based and paper versions during school time with an interval of at least 2 h between administrations. The order of administration was randomized. Participants completed EQ-5D-Y, a measure of mental health status (the Strengths and Difficulties Questionnaire), and sociodemographic variables using a self-administered questionnaire. Parents questionnaire included parental level of education and presence of chronic conditions in children. Missing values, and floor and ceiling effects were compared between versions. Mean score differences were computed for the visual analogue scale (VAS). Percentage of agreement, kappa index (k) and intraclass correlation coefficient (ICC) were computed to analyze the level of agreement between web-based and paper versions on EQ-5D-Y dimensions and VAS. Known groups validity was analyzed and compared between the two formats.

**Results:**

Participation rate was 77 % (n = 715). Both formats of EQ-5D-Y showed low percentages of missing values (n = 2, and 4 to 9 for web and paper versions respectively), and a high ceiling effect by dimension (range from 79 % to 96 %). Percent agreement for EQ-5D-Y dimensions on the web and paper versions was acceptable (range 89 % to 97 %), and k ranged from 0.55 (0.48-0.61, usual activities dimension) to 0.75 (0.68-0.82, mobility dimension). Mean score difference on the VAS was 0.07, and the ICC for VAS scores on the two formats was 0.84 (0.82-0.86). Both formats showed acceptable ability to discriminate according to self-perceived health, reporting chronic conditions, and mental health status.

**Conclusions:**

The digital EQ-5D-Y showed almost identical VAS scores and acceptable levels of agreement on dimensions. Both formats demonstrated acceptable levels of construct validity. Availability of the Spanish and Catalan web-version will facilitate its use in HRQOL assessment and in economic evaluation.

## Background

Health-related quality of life (HRQOL) assessment in children and adolescents is an increasing area of interest in public health research and in daily clinical practice [1]. Systematic reviews have identified several generic and condition-specific instruments for use in children [2, 3]. However, one limitation of many of these instruments is their lack of correspondence and continuity with HRQOL instruments for use in adults, which makes it difficult to analyze changes in HRQOL using a life course approach.

One instrument which avoids this limitation to a large degree is the EQ-5D-Y, a variant of the EQ-5D instrument developed by the EuroQol group for use in children and adolescents from 8 to 18 years of age [4, 5]. The EQ-5D-Y was based on a revision of the standard version of the EQ-5D and has very similar content. It thereby provides continuity with the standard, adult version, and has also demonstrated its feasibility, reliability, and validity in the population for which it was designed. As with the standard EQ-5D, it is quick and easy to administer.

Patient reported outcomes (PROs) such as EQ-5D-Y are increasingly used in a variety of formats, ranging from the traditional self-administered pencil and paper format, to use on a range of electronic media including tablets, web versions, and PDAs [6]. While studies have shown that results obtained with electronic versions are generally similar or superior to pencil and paper versions, in terms of outcomes and psychometric properties, it can still be worthwhile to test equivalence between formats if significant changes are required to use the instrument in a different format [7] or if the instrument is designed for use in a population, such as children, in which a lot of equivalence testing has not been performed. The popularity and successful use of information and communication technologies (ICT) among younger populations [8–11], coupled with the greater facilities that electronic versions provide, makes it likely that PROs for children will be increasingly administered in digital format, in versions specifically designed for children and teenagers [12]. As far as we are aware, the equivalence of paper and electronic versions of EQ-5D-Y has not been investigated to date. The inclusion of Spanish and Catalan versions of the instrument in a large, school-based, prospective study in Catalonia, Spain, made it possible to do so. The objectives of the present study were therefore to develop the web-based Spanish and Catalan versions of the EuroQol 5D-Y (eEQ-5D-Y) and to compare scores and psychometric properties with the paper version. Our hypothesis was that the level of agreement between the web- and paper-based versions would be similar independently of the language version used and that students would be able to complete both versions with the same level of competence.

## Methods

### Sample selection and study design

The sample included students from 3rd to 6th course of Primary education (approximately 8 to 11 years old), 1st through 4th grade of Secondary education (12-16y), and High School (17-18y) from 3 schools in Palafolls, a town in the province of Barcelona with approximately 9000 inhabitants (n = 923). This convenience sample was selected in part because earlier contacts with teaching staff and parents would facilitate participation in the study. We included the whole school age population of the town in order to increase representativeness. All students whose parents previously signed consent to participate and who voluntarily agreed to participate were included in the study. Children/adolescents with a level of cognitive impairment that would compromise their understanding of the questionnaires were excluded from the study. Fieldwork was carried out between October and November, 2013.

All participants answered the paper and web-based versions on the same day with a minimum of 2 hrs between administration of the two formats. The interval between administrations was filled with a 1 h class and several distracting activities. Individuals were randomized to complete either the web or the paper version first. Given the bilingual characteristics of the study population, the two language versions used in the study (Spanish and Catalan) were also randomized between individuals, with each student answering the web and paper version of the same language.

All procedures were carried out following the data protection requirements of the European Parliament (Directive 95/46/EC of the European Parliament and of the Council of 24 October 1995 on the protection of individuals with regard to the processing of personal data and on the free movement of such data). The ethical and legal requirements in Spain were also adhered to, and the protocol was approved by the Clinical Research Ethics Committee of the Hospital del Mar, Barcelona.

### Instruments and variables

The EQ-5D-Y was developed from the EQ-5D by adapting the original questionnaire to the requirements of measuring HRQOL in children and adolescents from 8 years onwards [4, 5]. It consists of a descriptive system covering 5 dimensions of health, i.e. mobility (‘walking about’), self-care (‘looking after myself’), usual activities (‘doing usual activities’), pain and discomfort (‘having pain or discomfort’), and anxiety and depression (‘feeling worried, sad or unhappy’). In each dimension, respondents are asked to rate their health ‘today’ on one of three levels (‘no problems’, ‘some problems’ and ‘a lot of problems’). The EQ-5D-Y also includes a vertical, graduated Visual Analogue Scale (VAS), on which the respondent rates his/her overall health on the day of the interview on a scale from 0 and 100, with 0 representing the worst and 100 the best health state he/she can imagine.

The web-based version of the questionnaire was developed using Ruby on Rails applications and the MySQL database (http://rubyonrails.org). Our version can be used on tablets but not on smart phones or other small devices. This version was developed following advices from the Euroqol group and followed the paper-based format as far as possible. All five dimensions were presented on one page with a further page for the VAS. Students were not able to scroll back to correct or modify the answer once they finished the questionnaire. Moreover a warning appeared before logging out if there were one or more missing answers, and no more than one answer was possible for each dimension. The research group carried out a pilot test which included approximately 20 simulations to detect inconsistencies, errors, and problems with the use of the digital version.

The Spanish and Catalan versions of EQ-5D-Y were produced following EuroQol Group guidelines [13–15], which require two independent forward translations, back translation, and cognitive debriefing in 8 members of the target population. Children's mental health status was assessed using the Strengths and Difficulties Questionnaire (SDQ), a brief behavioural screening questionnaire for children and adolescents that asks about their mental health symptoms and positive attitudes [16]. The instrument consists of 25 items measuring 5 dimensions of emotional symptoms, conduct problems, hyperactivity/inattention, peer relationship problems, and pro-social behaviour. All items are scored on a three point scale with 0 = ‘not true’, 1 = ‘somewhat true’, and 2 = ‘certainly true’. Higher scores indicate more problems except on the pro-social behaviour dimension. Items in the 4 problem dimensions are summed to give a total difficulties score ranging from 0 (no problems) – 40 (maximum problems). The Spanish version has been shown to be reliable and valid [17]. Other variables collected included age, sex, self-rated health, family socio-economic status, family type, and parental level of education. Socio-economic status was measured using the Family Affluence Scale (FAS) [18], which covers family car ownership, whether children have their own unshared room, the number of computers at home, and time spent on holiday in the previous 12 months. FAS scores were categorized as ‘low’ (0–3), ‘intermediate’ (4–5), and ‘high’ (6–7) affluence level. Self-rated health was categorized as ‘excellent’, ‘very good’, or ‘good’ versus ‘fair’ and ‘poor’.

Additionally, parents answered a questionnaire to provide information on the highest family level of education (primary, secondary, university) and whether their child had any chronic health problems (yes/no) from a list of 16 common conditions, such as asthma, diabetes, epilepsy, etc.

### Statistical analysis

The feasibility and acceptability of eEQ-5D-Y was investigated by calculating the percentage of missing values on the descriptive system and VAS compared with the paper version.

Percent agreement and kappa coefficients [19] were used to estimate concordance between modes of administration. The intraclass correlation coefficient (ICC) [20] was used to assess agreement between scores on the paper and web-based versions of the VAS. Kappa values were interpreted according to Landis and Koch’s guidelines [21] with kappa <0.2 indicating poor agreement, 0.21–0.40 indicating fair agreement, 0.41–0.60 moderate agreement, 0.61–0.80 substantial agreement, and kappa 0.81 or higher indicating almost perfect agreement. An ICC over 0.7 was considered as acceptable. Bland-Altman plots and the 95 % confidence interval (95 % CI) were also calculated to determine upper and lower limits of agreement for the VAS [22]. Finally, the prevalence of different EQ-5D-Y health profiles, based on the pattern of responses to each item, were explored and the number of profiles obtained with each version and the characteristics of the more prevalent profiles compared.

Validity was examined by assessing the known groups’ validity of the EQ-5D-Y. This involved comparing the results on the descriptive system and the VAS of the paper- and web-based versions to determine whether they both discriminated between groups which were a priori expected to show differences in HRQOL, i.e. according to self-perceived health, the presence of chronic conditions and mental health status scores obtained from the SDQ questionnaire. Comparisons were performed using Chi square tests and the categories of ‘some’ and ‘a lot of problems’ were collapsed to one category (‘any problems’). *T*-test or ANOVA were used to assess the relationships between VAS and perceived health, reporting chronic conditions, and mental health. It was hypothesized that the dimension of anxiety/depression (‘feeling worried, sad or unhappy’) would show a moderate to high correlation with the SDQ total difficulties score and that similar correlations would be seen between the VAS and the general health item, the number of the chronic conditions reported by parents, and mental health status.

Data was analyzed using the SPSS v. 18 software (SPSS Inc., Chicago, USA).

## Results

Minor changes were made to the internet version after the pilot test to facilitate screen visualization. The participation rate in the school survey was 77 % (n = 715); one subject was excluded after survey administration due to cognitive impairment that prevented understanding of the questionnaire. Table 1 shows the sample’s sociodemographic characteristics. Forty-eight percent were in primary education, 54 % were girls, mean age was 11.7 years, 23 % were from families with at least one university degree, and 30 % were in the low FAS category. 49.5 % of the sample answered the Spanish version of the questionnaire. The average time to complete the web version was 1.25 mins; it was not possible to collect data on completion times for the paper version. There were no significant differences in terms of socio-demographic characteristics between students randomized to complete the paper then the web version first and those in the group that completed the web version first.

**Table 1:** Sociodemographic characteristics of the sample

	*n*	mean (s.d.) or %
**Age**		
Mean (SD)	713	11.7 (2.8)
7-11	339	47.5
12-15	289	40.5
≥16	85	11.9
**Sex**		
Female	385	54.0
Male	328	46.0
**Family type**		
Biparental	573	84.0
Monoparental	109	16.0
**Highest family level of education**		
Primary	163	25.1
Secondary	334	51.4
University degree	153	23.5
**Family Affluence Scale**		
Low	214	30.7
Middle	446	64.0
High	37	5.3
**Order of administration**		
Paper version first	375	52.6
Web version first	338	47.4
**Language of questionnaires**		
Spanish	353	49.5
Catalan	360	50.5

Missing values: age (2); sex (2); type of family (33); family affluence scale (18); level of education (65); order of administration (2)

*s.d.* standard deviation

### Feasibility

The percentage of missing values for the EQ-5D-Y was similarly low in both formats (n = 2 for all dimensions on the web version, n = 4 for all dimensions on the paper version except ‘doing usual activities’, n = 5, and n = 9 for the VAS in both versions). Table 2 shows the distribution of EQ-5D-Y scores. There was a similar percentage of responses in both formats in the ‘no problems’ categories. The dimensions with the highest rates of health problems (‘some/a lot’) in both formats were the ‘pain/discomfort’ and ‘anxiety/depression’ dimensions. The lowest rate of self-reported problems was on the self-care (‘looking after myself’) dimension, which showed a similar distribution of responses in both formats.

**Table 2:** Description of health states and VAS scores on the web-based and paper versions

	Web version	Paper version
	*n* (%)	*n* (%)
**EQ-5D-Y**		
**Mobility (walking about)**		
No problems	665 (93.3)	663 (93.3)
Some problems	45 (6.3)	45 (6.3)
A lot of problems	3 (0.4)	3(0.4)
**Looking after myself**		
No problems	691 (96.9)	686 (96.5)
Some problems	17 (2.4)	19 (2.7)
A lot of problems	5 (0.7)	6 (0.8)
**Doing usual activities**		
No problems	666 (93.4)	663 (93.4)
Some problems	40 (5.6)	42 (5.9)
A lot of problems	7 (1.0)	5 (0.7)
**Having pain or discomfort**		
No problems	569 (79.6)	575 (80.9)
Some problems	131 (18.3)	129 (18.1)
A lot of problems	13 (1.8)	7 (1.0)
**Feeling worried, sad or unhappy**		
No problems	574 (80.5)	570 (80.2)
Some problems	124 (17.4)	128 (18.0)
A lot of problems	15 (2.1)	13 (1.8)
**VAS**		
	**mean (s.d.)**	**mean (s.d.)**
	85.55 (16.5)	85.63 (16.2)

Distribution of the percentages of reported problems in each dimension of the questionnaire and VAS scores (0 = worst heath state, 100 = best heath state)

*VAS* Visual Analogue Scale, *s.d.* standard deviation

The ceiling effect was high and similar in both versions (from 96.9 % in ‘looking after myself’ to 79.6 % in ‘pain’ and 80.5 % in ‘feeling worried’ for the web version, and 96.5 % (‘looking after myself’) to 80.2 % (‘feeling worried’) for the paper version.

A total of 41 EQ-5D-Y health profiles were reported with the web-based version compared to 40 for the paper version. Table 3 shows a comparison of those health profiles with a reported prevalence ≥1 % on both questionnaire formats. The four most prevalent profiles were identical in both cases (11111, 11112, 11121, 11122). The best possible health state (11111) was reported by 66.3 % of respondents (n = 473) on the web-version, and by 65.0 % of respondents (n = 462) with the paper version; the worst possible health state (33333) was reported by 0.1 % in both versions (n = 1).

**Table 3:** Distribution of the reported health states with a prevalence ≥ 1 % for web-based and paper versions

Web version	*n* (%)	Paper version	*n* (%)
11111	473 (66.3)	11111	462 (65.0)
11112	59 (8.3)	11112	64 (9.0)
11121	53 (7.4)	11121	46 (6.5)
11122	32 (4.5)	11122	39 (5.5)
21111	13 (1.8)	11211	12 (1.7)
21121	10 (1.4)	21111	11 (1.5)
11222	7 (1.0)	21121	10 (1.4)
		12111	9 (1.3)

The numbers correspond to different levels on each of the EQ-5D-Y dimensions (mobility, self-care usual activities, pain and discomfort, anxiety and depression). Each number represents the reported problem for each dimension (1 = no problem, 2 = some problem, 3 = a lot of problems). Thus, 11223 means no problems in ‘mobility’ and ‘self-care’, some problems in ‘usual activities’ and ‘pain/discomfort’, and a lot of problems in ‘anxiety/depression’

### Agreement between versions

**Fig. 1:**
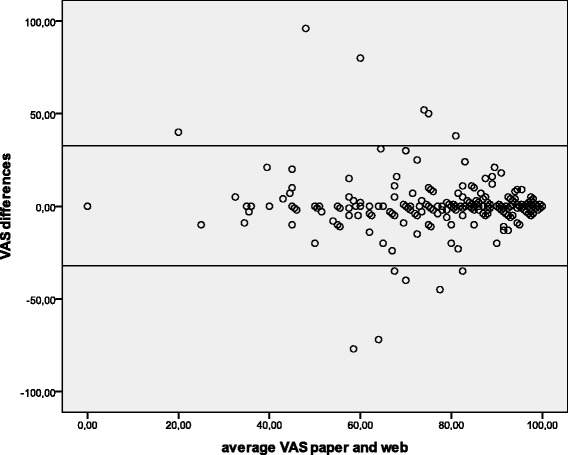
Bland Altman plot of the Visual Analogue Scale (VAS) web-based and paper versions

Percent agreement for EQ-5D-Y dimensions between the two formats was acceptable (range 89 % to 97 %). Kappa coefficients ranged from 0.55 (0.48-0.61, for ‘doing usual activities’ dimension) to 0.75 (0.68-0.82, for the ‘mobility’ dimension) (Table 4). The mean score difference on the VAS was 0.07, with the paper format giving a little bit higher scores and the ICC was 0.84 (0.82-0.86). Bland-Altman plots showed a slightly higher agreement for better VAS scores (Fig. 1), and the lower and upper limits of agreement were −17.38 to −18.51, and 17.52 to 18.65.

**Table 4:** Agreement between web-based and paper versions of the EQ-5D-Y dimensions and VAS

	Agreement (%)	Kappa (95%CI)
Mobility	97.0	0.75 (0.68-0.82)
Looking after myself	98.2	0.67 (0.60-0.73)
Doing usual activities	94.1	0.55 (0.48-0.61)
Having pain or discomfort	91.3	0.72 (0.65-0.79)
Feeling worried, sad or unhappy	89.5	0.65 (0.58-0.72)
	Mean difference (paper – web)	ICC (95%CI)
VAS	0.07 (9.01)	0.84 (0.82-0.86)

*VAS* Visual Analogue Scale, *ICC* Intraclass Correlation Coefficient, *CI* confidence interval

### Validity

Children reporting ‘fair’ or ‘poor’ self-perceived health on the general health item were more likely to report ‘some/a lot’ of problems on both formats than those reporting ‘excellent, very good, or good’ health (Table 5). Differences were statistically significant for all EQ-5D-Y dimensions except ‘looking after myself’ (Table 5). Those with higher (worse) SDQ scores were also more likely to report ‘some/a lot of’ problems (Table 5). The VAS showed a decreasing gradient according to the number of reported chronic conditions and results were similar for both formats (Table 6). Additionally, stratifying the data on age groups we observed a decreasing gradient in VAS scores according to age, and no significant differences in the dimensions except in ‘pain and discomfort’ (data not shown).

**Table 5:** Comparison of construct validity between web-based and paper versions of the EQ-5D-Y

	Web version		Paper version		Web version			Paper version		
	Self-perceived health (fair-poor) *n*(%)	P	Self-perceived health (fair-poor) *n* (%)	P	SDQ *n*	Mean (s.d.)	T	SDQ *n*	Mean (s.d.)	T
Mobility (walking about)		0.003		<0.001			<0.001			<0.001
No	25 (3.8)		18 (2.7)		649	8.9 (5.6)		645	8.9 (5.6)	
Some/a lot of	6 (12.5)		6 (13.6)		43	12.7 (6.1)		46	12.7 (6.2)	
Looking after myself		0.21		0.79			0.009			0.009
No	29 (4.2)		23 (3.4)		672	9.0 (5.6)		669	9.0 (5.6)	
Some /a lot of	2 (9.0)		1 (5.6)		20	11.7 (6.3)		22	12.3 (6.4)	
Doing usual activities		<0.001		<0.001			<0.001			<0.001
No	23 (3.5)		14 (2.1)		644	8.8 (5.6)		644	8.8 (5.4)	
Some/a lot of	8 (17.0)		10 (22.2)		46	14.1 (6.1)		45	14.1 (6.8)	
Having pain or discomfort		<0.001		<0.001			<0.001			<0.001
No	18 (3.2)		11 (1.9)		557	8.3 (5.3)		561	8.3 (5.4)	
Some/a lot of	13 (9.0)		13 (9.6)		137	12.6 (5.8)		130	12.5 (5.5)	
Feeling worried, sad or unhappy		<0.001		<0.001			<0.001			<0.001
No	16 (2.8)		8 (1.4)		558	8.3 (5.3)		553	8.1 (5.2)	
Some/a lot of	15 (10.7)		16 (11.5)		134	12.5 (5.9)		138	13.3 (5.6)	

Self perceived health (‘excellent’, ‘very good’, ‘good’ vs ‘fair’,’poor’)

*SDQ* Strengths and Difficulties Questionnaire

**Table 6:** Comparison of VAS scores between web-based and paper versions according to the number of reported chronic conditions

	Web version *n*	mean (s.d.)	Paper version *n*	mean (s.d.)
**Chronic conditions**				
**No**	439	87.36 (16.1)^a^	439	87.6 (15.0)^b^
**1**	168	84.31 (15.9)^a^	168	83.4 (16.7)^b^
**2**	63	81.9 (16.5)	63	82.0 (17.6)
**3 or more**	34	75.4 (20.6)^a^	34	76.5 (20.0)^b^

Chronic conditions reported by parents

*VAS* Visual Analogue Scale

^a, b^Statistically significant differences according to post-hoc comparisons using Bonferroni post-hoc test. Missing value chronic conditions: 11

Reliability and validity coefficients showed no statistically significant differences when stratifying the sample by language version (i.e. Catalan VAS mean = 84.5; Spanish VAS mean = 86.5; Effect size = 0.12). It is also of note that there was no evidence of improvement in scores on the second administration.

## Discussion

The present study is the first to assess the equivalence between the digital and papers formats of the EQ-5D-Y and to compare the known groups’ validity of the two formats. The results show that the eEQ-5D-Y is feasible and that it has an acceptable level of agreement with the paper version. Both formats demonstrated similar and acceptable known groups’ validity.

The development of a computer-based questionnaire offers a number of advantages but also some limitations compared with a paper-and-pencil version. Missing values or incomplete data can be reduced by requiring completion of an item before the individual can move on to the next question, although in the present study very few missing values were seen in either the web-based or paper formats. Out-of-range values can also be dealt with before reaching the stage of data checking and analysis, though this aspect is likely not so relevant with the EQ-5D-Y questionnaire. Furthermore, web-based versions cut down on the amount of time spent entering data and handling paper and data accuracy is increased by reducing typing or copying errors. Computer software can also score patient responses immediately and create summary information quickly for feedback to researchers and/or the respondents themselves. On the other hand, use of paper versions means that all items are visible all the time, while usually only one item or a set of items is visible at a time on the computer screen. When completing the paper version, one can gain an overall impression of the questionnaire before choosing the appropriate option for each item. In addition, paper versions provide an opportunity to change/correct responses if the subject so chooses. Digital versions are usually programmed in such a way that respondents cannot go back to change their previous answers.

The results of the present study confirm those of earlier studies which demonstrated that online health questionnaires are feasible in younger populations, especially among adolescents [23], and that different formats lead to comparable scores and show similar psychometric properties [24]. A meta-analysis concluded that there was extensive evidence to show that paper and computer versions of self-reported questionnaires are equivalent [25]. The present study reinforces those results as we found very few missing responses on the web-based version and very high agreement between the two formats. Future research with the web-based version should also focus on its use in groups which might represent a particular challenge, such as children with learning difficulties. It would also be of interest to examine how well a web-based version can be used in clinical practice.

### Feasibility

There was a low rate of missing values on both formats in this study, and we did not observe any problems with understanding of the EQ-5D-Y, either on the dimensions or the VAS. The rate of missing values was a slightly higher for the paper version than for the web-based version, as was expected based on previous studies [7]. This may have been due to the missing response alert feature on the web-based version. Nevertheless, overall, these figures indicate a very negligible rate of missing responses on both formats, at 0.002 % of missing values for the paper version and 0.005 % for the web-based version, and approximately 1 % for the VAS. This is encouraging for potential users of either format of the instrument. The slightly higher rate of missing responses on the VAS may have been due to it being a somewhat more cognitively demanding task, but we were not able to explore the issue further. This highlights the feasibility of EQ-5D-Y for use in younger populations, as reported earlier [5]. The results of the present study show a similar distribution of scores using both versions of the questionnaire and a similar pattern of scores to those found in the original validation study [5]; the high ceiling effect (particularly in the ‘looking after myself’ dimension) may be one of the instrument’s limitations for use in relatively healthy populations. Although the high ceiling effect may limit the ability of EQ-5D-Y to detect moderate impairments of HRQOL, it should be noted that both formats tested here were able to satisfactorily discriminate between groups categorized by their health status on other measures. An expanded number of response choices may also help to improve the instrument’s discriminatory capacity and sensitivity, as in the case of the adult version [26, 27], and is something the EuroQol Group is currently working on.

### Agreement between versions

The present study showed an acceptable level of agreement between both formats with moderate to substantial agreement in terms of percent agreement and kappa coefficients for the dimensions and acceptable ICC values for agreement on the VAS. Similar levels of agreement have been observed in comparisons of digital and paper versions of other instruments for use in younger populations [24].

### Validity

As noted, the ability of the EQ-5D-Y to discriminate between groups defined by their health status on other measures was acceptable and similar in both formats of the questionnaire. The results support the validity of all EQ-5D-Y dimensions, with the exception of the ‘Looking after myself’ dimension. However, the number of respondents reporting problems of any sort on this dimension was miniscule which would make discriminating between groups difficult. These results were similar to that of a previous study [5].

The Spanish and Catalan language versions of the instrument also showed similar and acceptable reliability and validity coefficients, with minor differences on reliability coefficients. We considered it important to test the two language versions in this study, as the population of Catalonia is largely bilingual and it is important to be able to offer both language versions for use in surveys there.

Some limitations of the study deserve comment. The sample selected in the present study to compare the web-based and paper versions included the whole school population of the town of Palafolls and may not be representative of that age group in Catalonia or Spain. Nevertheless, in this type of study, the samples do not need to be representative, though inclusion of respondents representing a broad range of health states will help to give a more accurate picture of the level of agreement; if too high a proportion of the sample is in a narrow range of health some of the correlation coefficients can be artificially inflated. In this case, although the sample was relatively healthy and the ceiling effect was correspondingly large, the large sample size meant that there was a reasonable spread of health characteristics. Secondly, the fact that both versions were administered on a single day may have led to some recall bias. This approach was used to minimize disruption in the school program. Although little information is available to indicate the extent to which the results of such equivalence studies are influenced by the interval between administrations, a study which compared the test-retest reliability of health status instruments using a 2-day or 2-week time frame between administrations found that the interval did not affect the results [28]. Other studies of equivalence between formats in children have used similar strategies to those employed here and found acceptable results [29]. On the other hand, a certain retest effect has been described, with improvements in the second administration independently of the time between measures [30]. In the present study, we randomized the order in which the web and paper versions were administered, and there was no evidence of improvement in scores in the second administration. Thirdly, each participant answered both paper and web-based version in the same language. The idea was to test both the Spanish and Catalan versions as both languages are widely used in Catalonia and we wanted to check that the level of agreement between web- and paper-based versions was similar in both languages. No differences were found when analyzing the results on EQ-5D-Y according to language, which likely reflects the bilingual characteristics of the sample and indicate a substantial level of agreement between the Catalan and Spanish versions of EQ-5D-Y. Finally, convergent validity was not assessed in the present study and should be incorporated into future studies of the Spanish and Catalan versions.

## Conclusions

The web-based and paper versions EQ-5D-Y provide comparable results on the profile and VAS in both Spanish and Catalan. The results of studies using the two different formats can therefore be compared and the two different forms of administration can be incorporated into the same study if necessary. Both formats demonstrated acceptable levels of construct validity. The web-based version of EQ-5D-Y provides an attractive format for younger respondents, and is easier to manage than the paper version. Its availability in Spanish and Catalan will facilitate its use in HRQOL assessment and economic evaluations.
